# In vivo organoid growth monitoring by stimulated Raman histology

**DOI:** 10.1038/s44303-024-00019-1

**Published:** 2024-06-28

**Authors:** Barbara Sarri, Véronique Chevrier, Flora Poizat, Sandro Heuke, Florence Franchi, Louis De Franqueville, Eddy Traversari, Jean-Philippe Ratone, Fabrice Caillol, Yanis Dahel, Solène Hoibian, Marc Giovannini, Cécile de Chaisemartin, Romain Appay, Géraldine Guasch, Hervé Rigneault

**Affiliations:** 1 Aix Marseille Univ, CNRS, Centrale Med, Institut Fresnel, Marseille, France; 2 Ligthcore Technologies, Marseille, France; 3 CRCM, Inserm, CNRS, Institut Paoli-Calmettes, Aix-Marseille Univ, Epithelial Stem Cells and Cancer Lab, Marseille, France; 4 Department of Biopathology, Institut Paoli-Calmettes, Marseille, France; 5 Department of Surgical Oncology, Institut Paoli-Calmette, Marseille, France; 6 Department of Gastro-enterology, Institut Paoli-Calmettes, Marseille, France; 7 Aix- Marseille Univ, CNRS, Neurophysiopathology Institute, Marseille, France

**Keywords:** Multiphoton microscopy, Biomedical engineering, Cancer imaging, Cancer imaging

## Abstract

Patient-derived tumor organoids have emerged as a crucial tool for assessing the efficacy of chemotherapy and conducting preclinical drug screenings. However, the conventional histological investigation of these organoids necessitates their devitalization through fixation and slicing, limiting their utility to a single-time analysis. Here, we use stimulated Raman histology (SRH) to demonstrate non-destructive, label-free virtual staining of 3D organoids, while preserving their viability and growth. This novel approach provides contrast similar to conventional staining methods, allowing for the continuous monitoring of organoids over time. Our results demonstrate that SRH transforms organoids from one-time use products into repeatable models, facilitating the efficient selection of effective drug combinations. This advancement holds promise for personalized cancer treatment, allowing for the dynamic assessment and optimization of chemotherapy treatments in patient-specific contexts.

## Introduction

Stimulated Raman histology (SRH) is an imaging technique based on stimulated Raman scattering (SRS)^1^, a non-linear optical microscopy technique that targets the vibrational signature of molecular bounds and enables label-free chemical imaging^2^. Imaging with SRS the CH_2_ and CH_3_ vibrational bounds in tissue samples allows us to highlight the distribution of lipid-rich content and protein–DNA-rich content, respectively, without any markers or labels^3–5^. When combined, these chemical images can be turned into virtually stained hematoxylin–eosin (HE)-like that can be used to perform a microscopic examination. SRH was applied to the central nervous system (CNS)^6,7^ and to the digestive system^8,9^. SRH demonstrated excellent agreement with standardly eosin and hematoxylin (HE) stained cryosection samples^10,11^ and, in some cases, can predict molecular alterations^12^. Lately, SRH applied on various CNS tumor samples has shown to be compatible with pathology workflow^13^ and appeared to be a useful tool for ‘in live’ tissue investigations^14^. In this paper, we present for the first time SRH applied to cancerous organoids called ‘tumoroids’, and we explore the feasibility of following such samples’ growth over time. We also extend SRH to 3D histology, using the unique ability of laser scanning SRS to perform virtual z sections of the sample. We demonstrate 3D virtual histology on tumoroids and in a gastric human biopsy sample.

Modeling a normal tissue or a particular pathology in vitro has opened perspectives for the development of personalized medicine where tissue replacement or drug discovery can be considered. Advances in stem cell biology have allowed the expansion of three-dimensional structures called organoids that display similar functional properties of the tissue of origin, including its genetic and cellular heterogeneity^15,16^. Stem cells present in the adult tissue can divide and differentiate properly into the organ of origin when put in the appropriate culture conditions that reflect their in vivo microenvironmental niche^17^. Organoids derived from tumor samples, also called tumoroids, can be derived, expanded and cryopreserved, and the resulting biobanks represent a wide range of spectrum of different subtypes of cancers. Indeed, the development of biobanks of tumoroids from various cancers, including hepatocarcinoma^18^, pancreatic ductal adenocarcinoma^19^, representation of the most common subtypes of stomach cancers^20^ and various grades and subtypes of colorectal cancers^21,22^, offer a model of choice for testing the efficiency of a large library of the drug to identify potential active agents specifically on the tumor of interest^23^. Organoids can also be derived from the normal adjacent tissue, allowing the identification of new drugs that specifically target tumor cells without damaging healthy cells^18^. These tiny organoids, ranging from micrometer to millimeter scale, have become a model of choice for many applications, but their small sizes are often a technical challenge for their histological analysis using classic embedding methods such as paraffin. Some improvement techniques have been proposed to obtain better histology, including the use of agarose as a pre-embedding step to concentrate the organoids^21,24^, but with these experiments, the organoids are fixed and cannot be reused later on for viable assays. Here we demonstrated the potential of stimulated Raman histology (SRH) on rectal cancer patients-derived organoids to provide a three-dimensional histopathology without any perturbation of the biological functionality of the living cells. With this technology we demonstrate the possibility to follow the growth, at the histological level, of the same organoid over ten days in culture. These results open promising perspectives to follow the effect of drugs at the cellular level while keeping the organoids alive. We also extend SRH to the 3D virtual histology of a human gastric biopsy sample and report 3D virtual HES over mm^2^ field of view and ∼100 µm depth with sub-cellular resolution.

## Results

**Fig. 1:**
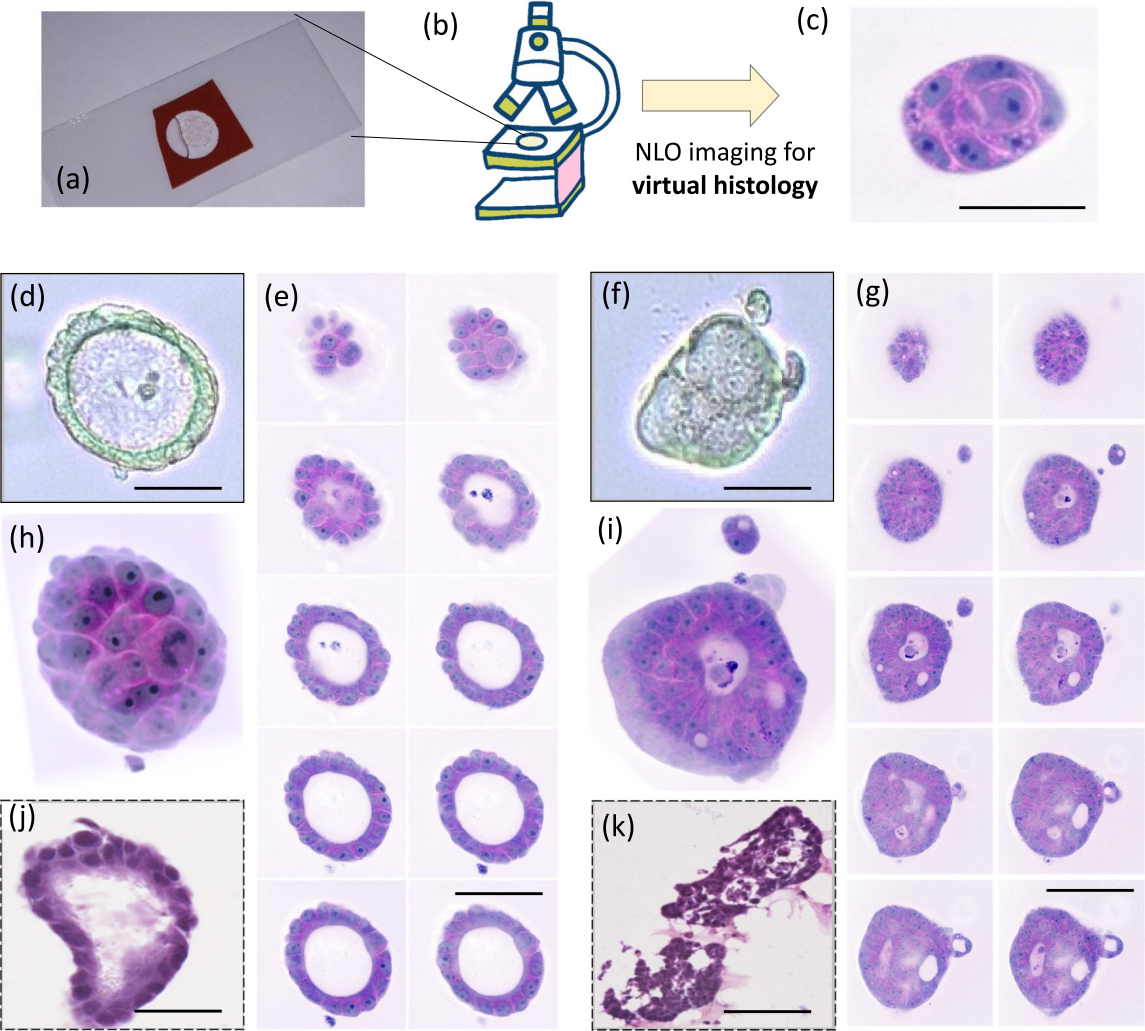
Stimulated Raman histology (SRH) applied to organoids. **a** A matrigel droplet is mounted between 2 coverslips and placed onto the SRH microscope (**b**) for virtual histology imaging (**c**). **d** Phase contrast image of cancerous organoids looking like a normal rectum organoid (scale bar = 75 µm), **e** SRH images of the same normal-like organoid acquired at different depths: z step between each image is 5 µm, scale bar = 150 µm, **f** Phase contrast image of a cancerous organoid (from human rectum), scale bar = 75 µm, **g** SRH images of the same cancerous organoid acquired at different depths: z step between each image is 5 µm, scale bar = 150 µm, **h** 3D-reconstruction of the normal-like organoid from SRH images presented in (e), **i** 3D-reconstruction of the cancerous organoid cut halfway through from SRH images presented in (**g**), (**j**) HE image of a normal-like rectum organoid from the same patient, scale bar = 100 µm, **k** HE image of a cancerous rectum organoid from the same patient, Scale bar = 100 µm.

The tumoroids were produced from a biopsy of human rectal mucosa infiltrated by adenocarcinoma with probable mucinous contingent (see the “Methods” section). The culture was composed of a heterogeneous population of organoids, with some presenting a normal-like morphology with spherical shapes constituted of monolayers of organized cells and a lumen (Fig. 1d) and others more disorganized (Fig. 1f). These two types of tumoroids were simultaneously images by SRS at the CH_2_ and CH_3_ chemical bonds (Supplementary Information movies M0 and M1) and instantaneously processed in SRH^14^ (see the “Methods” section). In contrast to the poor cellular architecture maintenance of the organoids obtained after conventional cryo-sectioning and HE staining (Fig. Fig. 1j, k), each single cell composing the tumoroids can be analyzed in detail by SRH. Figure Fig. 1e–g shows different SRH virtual cross-sections (every 5 µm) of the organoids (shown in Fig. Fig. 1d–f). Note that these sections are obtained by simply translating the microscope objective along the *z*-axis, as SRS imaging provides natural *z*-sectioning capability where the laser beams are focused and scanned. One can clearly see cell nuclei content as well as cytoplasmic structure evolution with depth. Moreover, 3D reconstruction of the collected images (Fig. 1c, h, i) allows a complete analysis of the entire tumoroids without losing any sections (see Supplementary Information Movies M2 and M3). The cell walls are particularly visible. Around 50 organoids of different sizes and shapes could be imaged in SRH (Supplementary Information S1 and T2) with the same quality during the 8–10 runs performed. We could observe that the organoid growth is unperturbed by SRH. Every time SRH could be successfully applied to tumoroids and offered a complete histological analysis of all the live cells of the tumoroids compared to traditional histopathological procedures.

**Fig. 2:**
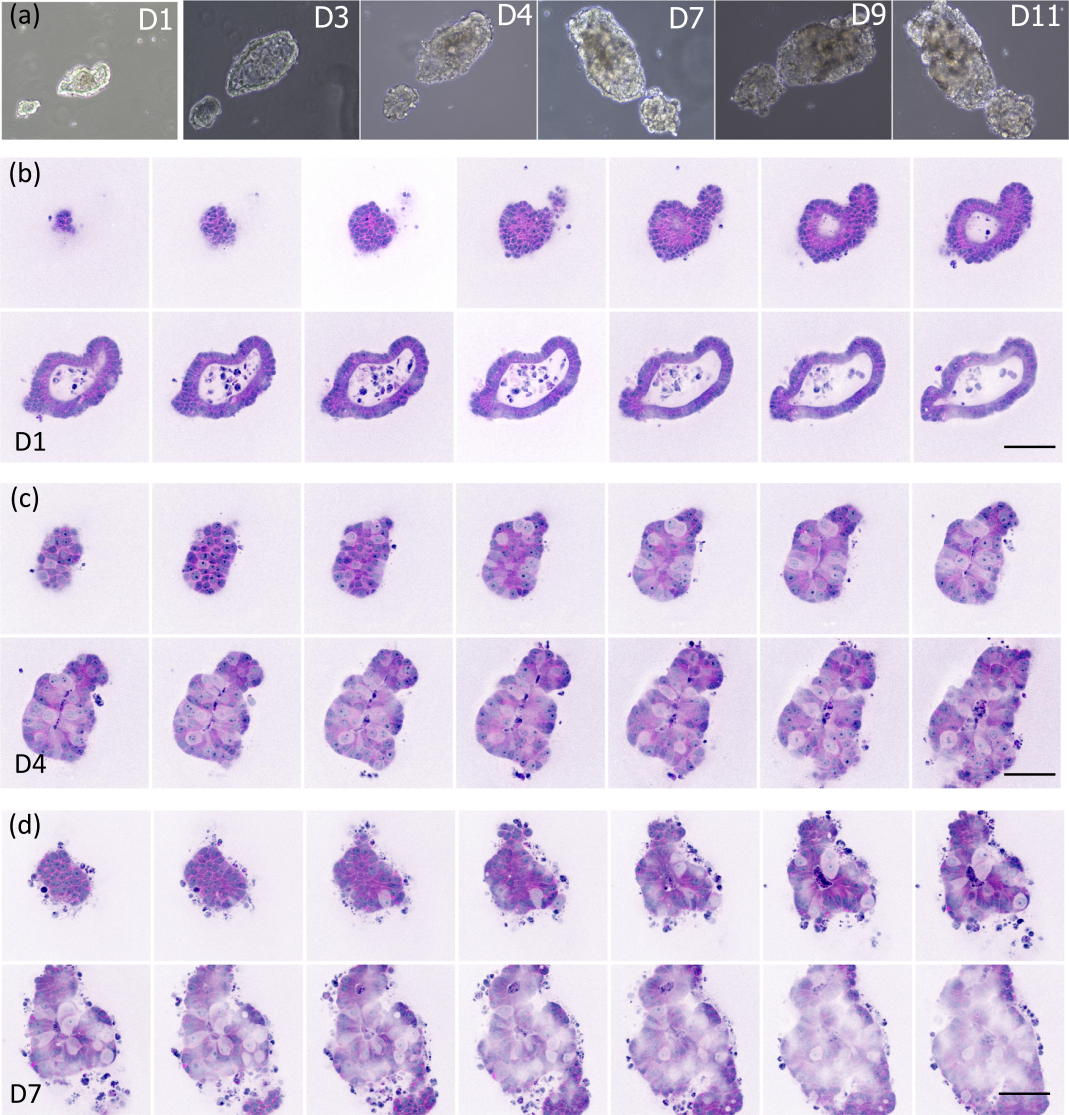
Organoid growth monitoring in SRH: From normal-like organoid to cancerous one. **a** Phase contrast images of an organoid imaged on different days—during the experiment (day 1 (D1), day 3 (D3), day 4 (D4), day 7 (D7), day 9 (D9), and day 11 (D11). **b** SRH images of the same organoid at D1 acquired at different depths, z step = 4 µm, **c** SRH images of the same organoid at D4 acquired at different depths, z step = 6 µm, **d** SRH images of the same organoid at D7 acquired at different depths, z step = 8 µm. Scale bar = 200 µm. Total power at the sample plane: 90 mW.

**Fig. 3:**
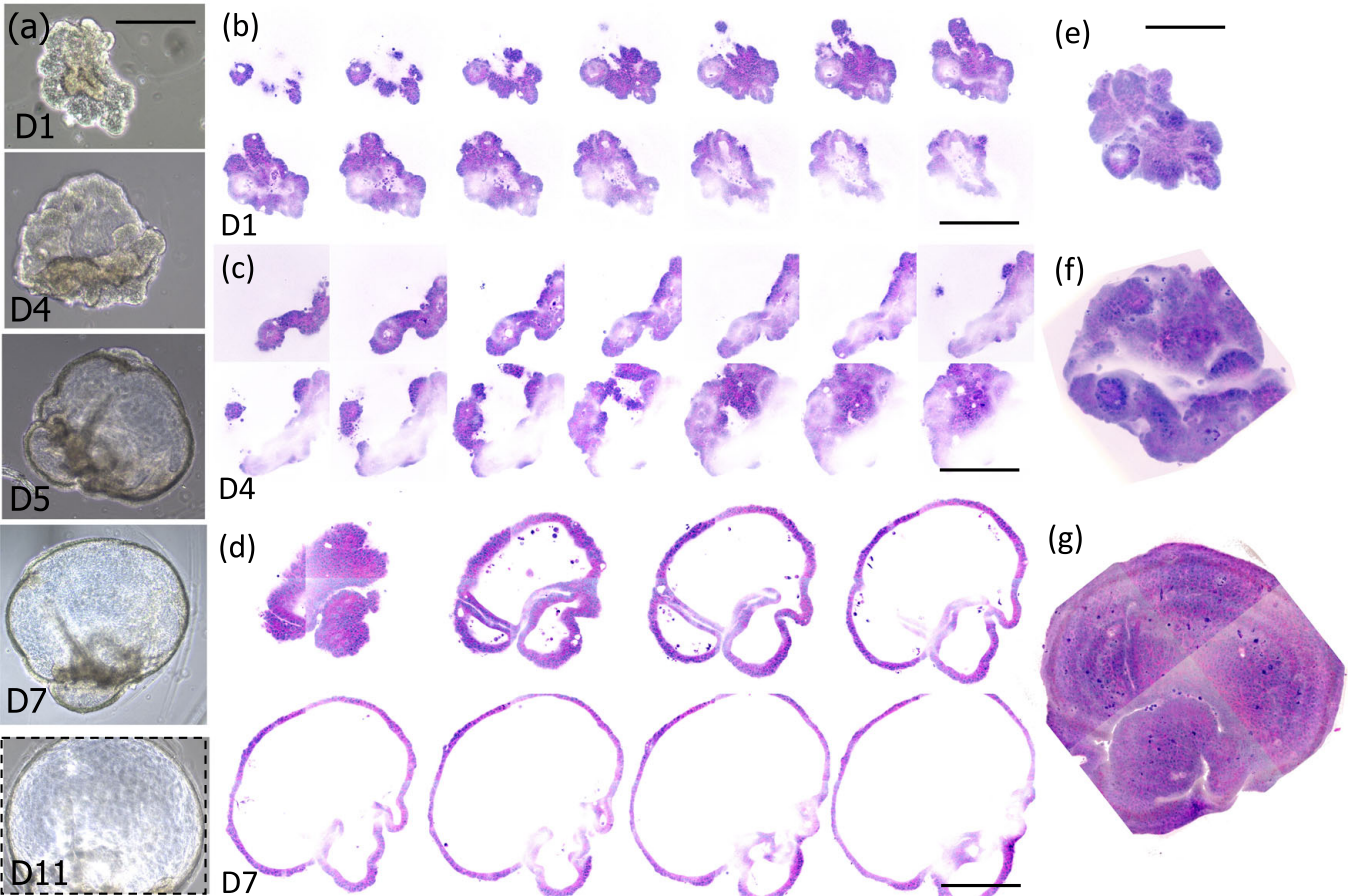
Organoid growth monitoring in SRH: From undifferentiated organoids to normal-like organoids. **a** Phase contrast images of the organoid imaged on different days (day 1 (D1), day 4 (D4), day 5 (D5), day 7 (D7) and day 11 (D11)) of culture. **b** SRH images of the same organoid at D1 acquired at different depths, z step = 5 µm, scale bar = 200 µm, **c** SRH images of the same organoid at D4 acquired at different, z step = 8 µm, scale bar = 250 µm, **d** SRH images of the same organoid at D7 acquired at different depths, z step = 20 µm, scale bar = 300 µm, **e** 3D-reconstruction from the images acquired in **b** (D1), **f** 3D-reconstruction from the images acquired in **c** (D4), **g** 3D-reconstruction from the images acquired in **d** (D7). Phase contrast images, scale bar = 200 µm, 3D-reconstructions, scale bar = 100 µm. Total power at the sample plane: 90 mW.

Next, we wanted to test the feasibility of obtaining virtual histology on growing tumoroids by sequential imaging over 10 days of culture. We chose to follow tumoroids with specific shapes, contents, and evolutions as they constituted a demonstrative proof of concept. Figures 2 and 3 summarize the SRH results of growing organoids. They provide in-depth information—one SRH image every few microns—of virtual histological data acquired at day 1 (D1) (Figs. 2b, 3b), day 4 (D4) (Figs. 2c, 3c) and day 7 (D7) (Figs. 2d, 3d) of culture. Phase contrast snapshots of the same organoids are also presented for comparison (Figs. 2a and 3a) from D1 up to day 11 (D11) of culture.

The organoid in Fig. 2 appears with a layer of organized cells and possesses a lumen at D1, two characteristics of normal-like organoids (Fig. 2b and Supplementary Information M4). Nevertheless, the presence of numerous necrotic cells within the lumen forecasted its development into a cancerous organoid; the lumen disappearing over time to the benefit of disorganized cells presenting poor cytosolic content and numerous nucleoli at D4 and D7 (Fig. 2c, d and Supplementary Information movies M5 and M6).

On the contrary, the organoid shown in Fig. 3 had an initial size of around 200 µm at D1 and presented several asperities like intestinal villi (Fig. 3b). The progression in size and maturation is visible with a striking doubling size every three days (Fig. 3b–d, e–g and Supplementary Information M7–M9). In this case, the presence of necrotic cells in the middle did not prevent the tumoroid from evolving into a massive normal-like organoid with the formation of a large lumen surrounded by a thin layer of organized cells at D7 (Fig. 3d). Supplementary Information movies M7-M9 attest for the good histology quality of SRH virtual sections allowing for detailed cell content investigation of the organoid structure. Note that on D7 the size of the organoid (Fig. 3d) required the use of the mosaic-mode of the SRH microscope to grab the entire organoid morphology entirely (Supplementary information M9).

**Fig. 4:**
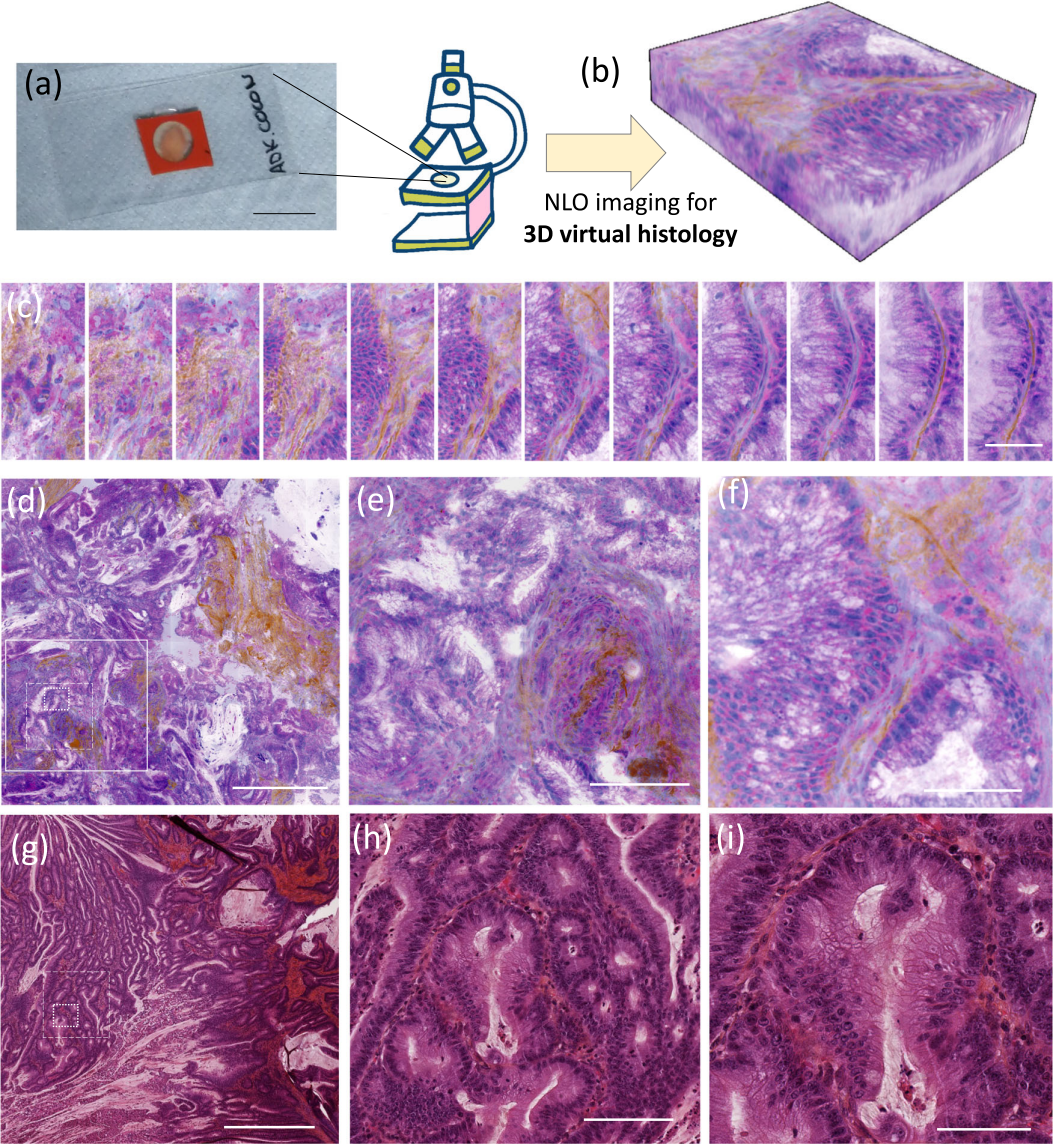
3D Stimulated Raman Histology (SRH) versus 2D standard eosin and hematoxylin safran (HES). **a** A fresh biopsy from surgery (here an adenocarcinoma (ADK)) is mounted between two coverslips and placed for examination on the BondXplorer SRH microscope to perform 3D histology, **b** SRH images at different z depths allow for 3D virtual histology reconstruction. Tissue investigation at both low and high magnifications is necessary to perform accurate diagnosis. **c** SRH images stack taken at a high magnification at different depths (z step = 2 µm), allow to investigate nuclei content in 3D and to follow the collagen distribution around the gland (scale bar = 20 µm), **d** SRH image from the biopsy at low magnification over a FoV of 2.1 mm × 2.1 mm (scale bar = 750 µm) enabling the identification of the tumorous gland deformations and collagen surrounding, **e** SRH image from the biopsy at medium magnification (FoV of 420 µm × 420 µm) allows to see the tumorous gland deformations more accurately (scale bar = 150 µm), **f** SRH image from the biopsy at high magnification (FoV 210 µm × 210 µm) (scale bar = 40 µm), **g** HES image of the ADK biopsy from the same patient at low magnification (scale bar = 750 µm), **h** HES image of the ADK biopsy from the same patient at medium magnification (scale bar = 150 µm, and **i** HES image of the ADK biopsy from the same patient at high magnification (scale bar = 40 µm). **e** and **f** are the magnified square ROIs delineated in long dash and dense dash in **d**, respectively. Total power at the sample plane: 90 mW.

We wish now to address the capability of SRH to image bulk tissue in 3D. This should allow the direct comparison of bulk tissue cell heterogeneity with their derived organoids. We investigate in Fig. 4 the 3D SRH of a human gastric adenocarcinoma (ADK) biopsy coming from surgery. The sample was mounted between 2 coverslips (Fig. 4a) and imaged using SRH (Fig. 4b–f). Standard histology HE from the same patient was performed in parallel for comparison (Fig. 4g–i). SRH acquisition conditions were chosen so that the time of each acquisition remained in the few minutes range to be compatible with intraoperative consultation (IC) conditions. We intentionally followed the same steps as during IC to mimic the pathologist’s examination. We first evaluated the general tissue architecture and parenchyma alteration at low magnification (FoV 2.1 mm × 2.1 mm) that allowed us to diagnose an infiltrative tumor with large gland deformations and a strong stroma (Fig. 4d–g and Supplementary information S2). Second, focusing on the region of interest at higher magnification (FoV of 420 µm × 420 µm*)*, the loss of the gland lumi and nuclei stratification could be seen (Fig. 4e–h). Finally, scrutinizing the cytoplasm and nuclei content at large magnification (FoV 210 µm × 210 µm) enabled to search for specific anomalies such as nucleioli (Fig. 4f–i). Analysis at each scale is of crucial importance for delivering an accurate diagnosis, here an adenocarcinoma. The depth and 3D reconstruction movies provided in supp. info allow both to appreciate the tissue architecture modification with depth and to follow the collagen fibers distribution around the glands (Supplementary information movies M10–M13).

## Discussion

Organoids derived from various tumors have been used to establish biobanks^25,26^, which provide invaluable tools to study individual responses to chemoradiation but also represent a preclinical model for drug screening^27^. One of the more important features that need to be validated when establishing biobanks is to reproduce the disease heterogeneity of the tumor of origin within the patient-derived organoids. In vivo, pathological analysis of the organoid compared to the tumor of origin is, therefore, an essential step. The rapid acquisition of histopathologic data on organoids while keeping them alive would be, therefore, very useful for daily screening of organoid cultures. In addition, the HE staining procedures used in hospitals are not always fitted for organoid coloring, resulting in over-colored HE sections sometimes hard to read. Fluorophores are largely used to study tumoroid behaviors. Getting access to label-free histology in parallel would free a fluorescence channel allowing multi-tag investigations. In this work, we have used SRH to image organoid structures without using any label or specific preparation, and we showed the potential of SRH to follow and characterize over time the growth and differentiation of rectal cancer patients-derived organoids, called tumoroids. The normal and cancerous tumoroids imaged by SRH (Figs. 1–3) in this study show good agreement with the HE-stained counterparts (Supplementary Information S3). This suggests that different cancer progression states can be detected by SRH. We used the z-sectioning ability of SRH to perform depth imaging and to provide 3D virtual histology of tumoroids. This allowed the analysis of the tissue 3D architecture with sub-cellular resolution while keeping the organoid intact and alive. It should be noted that the SRH imaging depth limit in a ‘bulk’ organoid, i.e. that is fully filled with cells, is around 150 µm (as can be seen in Supplementary Information movies M7 and M8) and that the ability to image in 3D organoids with a diameter >150 µm as shown in Fig. 3d is due to their empty lumen that reduces light scattering.

The power management is an important issue for time-lapse experiments as it is known that light exposure not only causes photobleaching of the sample but also alters cell physiology^28^. We have set the illumination power to 90 mW (25 mW@1025 nm, 40 mW@1034 nm, and 25 mW @793.5 nm) as a trade between pixel dwell time (dt = 10 µs) and SRS signal. With these parameters the 3D organoids could be imaged in ∼2 min and could develop over time. We found that local damages could be observed for a power >250 mW (dt = 10 µs).

Organoids are models of real tissues showing similar cellular heterogeneity and functional properties. To show that SRH can address this similarity, we have applied 3D SRH to a human gastric biopsy to enable HES virtual coloring over a depth of ∼100 µm. Recent developments in SRS deep tissue imaging using a Bessel beam could potentially improve this depth by two-fold^29^. This virtual SRH sectioning provides more detailed insights into the tissue 3D structure as compared to the few sections that are usually randomly prepared during conventional intra-operative consultation (IC) on frozen sections. The versatility and the rapid response time of the SRH microscope bring to the pathologist the ability to navigate through the sample in real time^14^ and to focus on a region of interest (ROI). We have shown that the SRH microscope enables mm^2^ investigation for large morphological tissue investigation but also a sub-cellular resolution that provides detailed cell nuclei analysis on a more limited (∼100 µm) field of view. Together with its tridimensional ability, SRH empowers the pathologist to rapidly detect and diagnose tumor progression to help in decision making during surgery, with an immediate benefit for the patient.

Other techniques have been reported to perform label-free virtual histology using artificial intelligence (AI) combined with auto-fluorescence^30^, UV photo-acoustic^31^, reflectance^32^, or multiphoton^33^ microscopy, to cite a few. Although those approaches have reported satisfactory results, they rely on contrast mechanisms that are not related to the chemical nature of the sample. Furthermore, none of these approaches can clearly identify cell nuclei that are always retrieved from the AI process. Due to its chemical sensitivity, virtual histology images based on SRS are not deduced from any AI processes but are generated directly from the chemical spatial differences between CH_2_ and CH_3_ images on solid physical ground. AI could directly benefit from the recent work that has shown an improvement of SRH images to the level of formalin-fixed and paraffin-embedded^34^.

For long-term organoid investigation, the SRH microscope could be equipped with an incubation chamber (37 °C, 5% CO_2_) to get access to live virtual HE to witness cell division and differentiation in real-time. With multi-well plates the SRH microscope would allow rapid multi-drug screening on tumoroids that could help in personalized medicine. Coupled with an automated organoid platform, SRH could be a valuable add-on and a possible game changer to advance personalized medicine^35^.

## Methods

### Culture of patient-derived organoids

Endoscopic human rectum biopsies are thinned in 2 mm pieces, washed twice in 1X PBS (14190094, Life-Technologie), and then dissociated 20 min at 37 °C under agitation in the digestion buffer (1 mL DMEM, 41965062, Life-Technologie + 2.5% fetal calf serum (SVF), F7524 Life-Technologie + 150 µL collagenase 20%, C2674, Sigma + 1 mL Dispase, 17105-041 Thermofisher). 12.5 µL of DNAse (89836, Thermofisher) are added for 10 min at 37 °C under agitation. The sample is resuspended in 50 mL of cold PBS and centrifuged for 5 min at 4 °C at 200 × *g*. The pellets are resuspended in 20 mL of PBS and then passed through a 70 µm filter (352350, Falcon). 200 μL SVF are added to the filtrate. The tissues or clusters of cells left on the filter were dissociated with 2 mL of Tryple Express (12605010, Life-Technologie) and incubated at 37 °C for 5 min. The action of trypsin is stopped by adding 9 mL of Complete AdDF Medium (Advanced DMEM/F12 + Glutamax + Penicillin) + 2.5% SVF. The mix is then passed on a 40 μm filter and centrifuged at 200 × *g* for 5 min at 4 °C. The pellet is resuspended in 1 mL Complete AdDF Medium, and the cells are counted. For the realization of tumor organoids, the cells are resuspended in matrigel (356231, Corning) in order to obtain a ratio of 1000–50,000 cells per drop of 25 μL matrigel. These drops are placed in the center of the wells of a 48-well culture plate previously heated to 37 °C. After a period of solidification of the matrigel for 10 min in an incubator at 37 °C 5% CO_2_, the drop is covered by 300 μL of organoid medium (Supplementary Information Table T1) supplemented by 10 μM of Y-27632 (during the first 5 days). After 7–10 days of culture, organoids are analyzed by SRH microscopy.

### Embedding and cryo-sectioning of tumoroids

Organoids were washed with PBS 1X and fixed in 4% paraformaldehyde (28908, Thermoscientific) overnight at 4 °C under agitation. The sample was then washed three times in PBS 1X and put in PBS 1X sucrose 30% overnight at 4 °C. Finally, organoids were incubated with 7.5% gelatin (G7765, Sigma) /15% sucrose (S7903, Sigma) in PBS 1X at 37 °C for at least 30 min and placed in mold to freeze at −80 °C. The slices (7–9 µm) are performed with cryostat (Cryostar NX70, Thermoscientific) at −30 °C and put on superfrost^TM^ plus adhesion microscope slides. The ICEP (Ipc Crcm Experimental Pathology) platform produces HES stains from samples included in gelatin–sucrose. The HES staining consists of hematoxylin dyeing the nuclei in violet, eosin dyeing the cytoplasm in pink, and Saffron dyeing the collagen in yellow.

### SRH imaging and 3D reconstruction

SRH imaging was performed using the BondXplorer™ SRS microscope (Lightcore Technologies) powered by the Delta-Emerald™ SRS source (APE), whose principle is detailed in ref. ^36^. The SRS signals from the CH_2_ and CH_3_ chemical bonds were detected simultaneously in the forward direction using a dual channel lock-in amplifier, while second harmonic generation (SHG) was detected at the same time in the backward (epi) direction by a photomultiplier tube. The Delta-Emerald™ provides two fixed beams at the 1034 and 1025 nm wavelengths and one tunable beam that can be set in 740–990 nm range. To image simultaneously the CH_2_ (2845 cm^−1^) and CH_3_ (2930 cm^−1^) chemical bonds the tunable wavelength is set to 793.5 nm. The three beams were focused at the sample plane with a ×25 magnification objective (Nikon, APO LW ×25/1.1w, wd), and the laser powers were set to 25, 40, and 25 mW for the 1025, 1034 nm, and tunable beam, respectively. The virtual SRH images are obtained in real-time by processing and coloring the CH_2_, CH_3_, and SHG images as presented in^14^. Different imaging modalities such as “Live”, “Time-Lapse”, “Depth Stack” or “2/3D Mosaic” could be chosen to acquire the SRH data. The pixel dwell time (dt) (illumination time spent per pixel), as well as the scan range (that sets the field of view (FoV)) and the resolution (number of pixels per fov), could be adjusted using the BondXplorer™ microscope interface software. For biopsies analyses, large-scale images (≃1mm^2^) could be recorded using the ‘2D Mosaic’ option, which stitches 210 µm × 210 µm laser-scanned images next to each other. The ‘Live’ mode allows the user to navigate in 3D within the sample quasi-instantaneously with a z-sectioning capability of a few microns^14^. In the following conditions: FoV 210 µm × 210 µm, 512 × 512 pixels, dt 10 µs, 2 µm z-spacing depth-stack; a 120 µm-thick organoid could be entirely investigated in less than 2 min 30 s. Virtual-HE 3D reconstructions were performed post-SRH imaging using the *Agave* software.

### Microscope sample mount

Organoids or biopsy samples were mounted between two coverslips separated by a 500 µm-thick spacer without further preparation other than adding a droplet of PBS (Figs. 1a and 4a). The sample could be recovered post-SRH imaging to re-enter into a pathology workflow or cell culture if needed.

### Time-lapse SRH imaging of organoid samples

Time-lapse SRH imaging of organoids was performed in 6 cm plastic Petri dishes whose bottom side had been modified to hold a 1-inch glass cover slip. This ensures optimal imaging using the high numerical aperture objective lens, eliminates the possible SRS noise coming from the plastic, and facilitates large displacements onto the sample, enabling the whole matrigel to be explored during SRH imaging sessions. For each modified Petri dish, an 18 mm diameter hole was made, and a 20 mm diameter cover slip was fixed using a biocompatible glue before sterilization. A matrigel droplet containing just a few organoids (~5–6) was then deposited in the middle of each modified Petri dish with 3 ml of feeding medium. Before each SRH imaging session, the feeding medium was washed and replaced with 37 °C PBS1X to allow for background-free SRS imaging. During SRH imaging sessions, (*x*,*y*,*z*) coordinates were used to locate each organoid within the matrigel from one session to another. This also allowed specific organoids to be distinguished from one another. Organoids were imaged using the z-stack option of the BondXplorer™ microscope, which provided depth screening within a few minutes per organoid. To prevent potential organoid photo-damage each SRH imaging session was limited to 30 min so that the entire routine never exceeded 45 min in total. Post-SRH imaging, PBS media was replaced by new 37 °C feeding media before re-entering the incubator. Organoid growth was also monitored daily using phase contrast microscopy in parallel. SRH imaging sessions were performed every 48–72 h.

## Supplementary Information

Supplementary Information

## Data Availability

The data that support the findings of this study are available on reasonable request from the corresponding author.
