# Author Correction: In vivo organoid growth monitoring by stimulated Raman histology

**DOI:** 10.1038/s44303-024-00035-1

**Published:** 2024-08-08

**Authors:** Barbara Sarri, Véronique Chevrier, Flora Poizat, Sandro Heuke, Florence Franchi, Louis De Franqueville, Eddy Traversari, Jean-Philippe Ratone, Fabrice Caillol, Yanis Dahel, Solène Hoibian, Marc Giovannini, Cécile de Chaisemartin, Romain Appay, Géraldine Guasch, Hervé Rigneault

**Affiliations:** 1 Aix Marseille Univ, CNRS, Centrale Med, Institut Fresnel, Marseille, France; 2 Ligthcore Technologies, Marseille, France; 3 CRCM, Inserm, CNRS, Institut Paoli-Calmettes, Aix-Marseille Univ, Epithelial Stem Cells and Cancer Lab, Marseille, France; 4 Department of Biopathology, Institut Paoli-Calmettes, Marseille, France; 5 Department of Surgical Oncology, Institut Paoli-Calmette, Marseille, France; 6 Department of Gastro-enterology, Institut Paoli-Calmettes, Marseille, France; 7 Aix- Marseille Univ, CNRS, Neurophysiopathology Institute, Marseille, France

**Keywords:** Multiphoton microscopy, Biomedical engineering, Cancer imaging, Cancer imaging

Correction to: *npj Imaging* 10.1038/s44303-024-00019-1, published online 28 June 2024

In this article, the affiliation details for authors Véronique Chevrier and Géraldine Guasch were incorrectly given as Centre de Recherche en cancérologie de Marseille, Marseille, France but should have been CRCM, Inserm, CNRS, Institut Paoli-Calmettes, Aix-Marseille Univ, Epithelial Stem Cells and Cancer Lab, Marseille, France. The affiliation details for authors Flora Poizat and Florence Franchi were incorrectly given as Institut Paoli-Calmettes, Endoscopy and Gastroenterology Departement, Marseille, France but should have been Department of Biopathology, Institut Paoli-Calmettes, Marseille, France. The affiliation details for author Eddy Traversari were incorrectly given as Centre de Recherche en Cancérologie de Marseille, Marseille, France but should have been Department of Surgical Oncology, Institut Paoli-Calmettes, Marseille, France. The affiliation details for authors Jean-Philippe Ratone, Fabrice Caillol, Yanis Dahel, Solène Hoibian, and Marc Giovanninni were incorrectly given as Institut Paoli-Calmettes, Endoscopy and Gastroenterology Departement, Marseille, France but should have been Department of Gastro-enterology, Institut Paoli-Calmettes, Marseille, France. The affiliation details for author Cécile De Chaisemartin were incorrectly given as Institut Paoli-Calmettes, Endoscopy and Gastroenterology Departement, Marseille, France but should have been Department of Surgical Oncology, Institut Paoli-Calmettes, Marseille, France. Additionally, the wrong Supplementary File was originally published with this article; it has now been replaced with the correct file. The original article has been corrected.

